# Red cell distribution width and common disease onsets in 240,477 healthy volunteers followed for up to 9 years

**DOI:** 10.1371/journal.pone.0203504

**Published:** 2018-09-13

**Authors:** Luke C. Pilling, Janice L. Atkins, George A. Kuchel, Luigi Ferrucci, David Melzer

**Affiliations:** 1 Epidemiology and Public Health Group, University of Exeter Medical School, Exeter, Devon, United Kingdom; 2 Center on Aging, University of Connecticut Health, Farmington, Connecticut, United States of America; 3 National Institute on Aging, Baltimore, Maryland, United States of America; National Yang-Ming University, TAIWAN

## Abstract

Higher Red Blood Cell Distribution Width (RDW or anisocytosis) predicts incident coronary artery disease (CAD) plus all-cause and cardiovascular mortality, but its predictive value for other common diseases in healthy volunteers is less clear. We aimed to determine the shorter and longer term associations between RDW and incident common conditions in participants free of baseline disease, followed for 9 years. We undertook a prospective analysis of RDW% using 240,477 healthy UK Biobank study volunteers aged 40–70 years at baseline, with outcomes ascertained during follow-up (≤9 years). Participants were free of anemia, CAD, type-2 diabetes, stroke, hypertension, COPD, and any cancer (except non-melanoma skin cancer) at baseline. Survival models (with competing Hazards) tested associations with outcomes from hospital admission records and death certificates. High RDW (≥15% variation, n = 6,050) compared to low (<12.5% n = 20,844) was strongly associated with mortality (HR 3.10: 95% CI 2.57 to 3.74), adjusted for age, sex, smoking status, education level, mean cell volume and hemoglobin concentration. Higher RDW was also associated with incident CAD (sub-HR 1.67: 1.40 to 1.99), heart failure, peripheral vascular disease, atrial fibrillation, stroke, and cancer (sHR 1.37: 1.21 to 1.55; colorectal cancer sHR 1.92: 1.36 to 2.72), especially leukemia (sHR 2.85: 1.63 to 4.97). Associations showed dose-response relationships, and RDW had long-term predictive value (≥4.5 years after assessment) for the majority of outcomes, which were similar in younger and older persons. In conclusion, higher RDW predicted onsets of a wide range of common conditions as well as mortality in a large healthy volunteer cohort. RDW is not just a short term predictor, as high levels were predictive 4.5 to 9 years after baseline in healthy volunteers. The wide range of outcomes reflects known RDW genetic influences, including diverse disease risks. RDW may be a useful clinical marker for inclusion in wellness assessments.

## Introduction

Red Blood Cells shed their nuclei and organelles during erythropoiesis, and have a unique biconcave shape for the efficient transport of oxygen in the bloodstream and released to tissues. Variation in Red Blood Cell (RBC) volume (RBC Distribution Width, RDW) increases with age [1] and is known to be raised in cardiovascular disease [2]. Greater RDW is also associated with increased risk of all-cause and cardiovascular mortality [3,4], and is predictive of survival in patients diagnosed with cancer [5]. The mechanisms proposed to explain the association of RDW with mortality include stressed erythropoiesis (rapid production of new RBC) and variations in RBC survival, both affecting the overall size distribution since young erythrocytes are larger than old ones [6]. Other causes of high RDW include anemia and other iron or folate deficiencies [7], dyslipidemia [8] and other metabolic abnormalities, and the pro-inflammatory state of aging [9]. We previously found that RDW has a considerable genetic component, with 29.3% of the variation in RDW attributable by common genetic variants [10]. Specific pathways now linked to RDW include lipid-related genetic loci, including the *APOE* locus implicated in Alzheimer’s disease, and genes related to iron homeostasis and telomere maintenance [10].

A relatively large study involving 8,513 participants free of CAD found that, independent of anemia, raised RDW (>12.6%) predicted coronary artery disease (CAD) mortality up to 6 years after assessment, and was a better predictor of mortality than C-reactive protein levels (CRP) [11]. However, whether RDW is a cause or consequence of underlying or undiagnosed diseases remained unclear [12]. The methods applied previously have been limited to Cox proportional hazards regression, known to overestimate the risk of death in analyses of specific diseases [13]; therefore more robust methods accounting for the competing risks of mortality are required.

We aimed to estimate associations between baseline RDW and incident disease and mortality over a 9 year follow-up, in a large healthy volunteer sample aged 40 to 70 at baseline. UK Biobank is likely the largest general population volunteer cohort in the world with such measures, with outcomes ascertained through hospital admission electronic records and death certificates. We excluded prevalent cases of CAD, anemia and other medical conditions, to focus on apparently healthy subjects. The current study expands on previous analyses by using a much larger sample (*n* = 240,244, compared to *n* = 8,175 and *n* = 11,827, respectively, for [3,4]) and excluding individuals with diagnosed disease at baseline. We also aimed to establish whether RDW’s predictive value was limited to the short term, or whether it remains predictive over longer periods. We also established whether RDW was a risk factor of mortality in relatively younger (40 to 59 years) individuals.

## Methods

The UK Biobank study includes 503,325 volunteers aged 40–70 years who were recruited by postal invitation to individuals living within 25 miles of the 22 assessment centers in Great Britain. The respondents (5.5% of the 9.2 million invited) were seen between 2006 and 2010, and data includes RBC distribution width (RDW) and other hematology measures, extensive questionnaires including smoking behavior and education history, and follow-up using electronic medical records. At baseline the participants were healthier than the general population, so the cohort may not be representative, however the results are likely to be generalizable [14].

We used data from 240,477 participants in our analyses after excluding those with the following prevalent diseases; anemia, coronary artery disease (CAD: myocardial infarction or angina), cancer, type-2 diabetes, stroke, chronic obstructive pulmonary disease (COPD) or hypertension. Participants provided informed consent for data linkage to hospital inpatient admissions, cancer registrations and death registrations. Ethical approval for UK Biobank study was obtained from the North West Multi-Centre Research Ethics Committee.

### RDW phenotype

RDW is a measure of the variability in the mean size of the RBC in each participant (in % units). It was measured using four Beckman Coulter LH750 instruments within 24 hours of blood draw, with extensive quality control performed by UK Biobank [15]. RDW is a continuous, highly skewed trait, so we divided the participants into 7 categories (<12.5%, 12.5–12.9%, 13.0–13.4%, 13.5–13.9%, 14.0–14.4%, 14.5–14.9%, ≥15.0%) comparable to previous analyses [4].

### Health outcomes

At the baseline interview participants were asked about prevalent diseases, including CAD and cancer. Participants were followed from baseline (2006–2010) using hospital episode statistics (HES) inpatient disease diagnosis (up to March 01, 2016), cancer registration (September 12, 2015) and death registration (August 15, 2015). In many cases, the records extend back before the interview date. Disease diagnoses were defined as either self-reported or hospital records, and exclusions were made for prevalent diseases at baseline in the analyses.

Anemia was determined both using self-reported diagnosis, electronic medical records (ICD10: D64* and D5* categories), or by low hemoglobin levels at the baseline assessment (<120g/L in females, <130g/L in males: from WHO definition [16]).

As for incident outcomes, we considered conditions likely to be reliably ascertained in hospital records: these included CAD (myocardial infarction or angina, *n* = 5,430), heart failure (*n* = 963), hospital diagnosed hypertension (*n* = 10,615), atrial fibrillation (*n* = 3,079), peripheral vascular disease (*n* = 728), and stroke (cerebral vascular disease, *n* = 1,390). We also investigated whether RDW% was associated with any incident cancer, excluding non-melanoma skin cancer (*n* = 11,486), and sub-types including breast (*n* = 2,323), colorectal (*n* = 1,327), prostate (*n* = 2,571), any type of blood cancer (*n* = 839), and specifically leukemia (*n* = 406) or lymphoma (*n* = 467; there were *n* = 38 individuals with both diagnoses in the follow-up and *n* = 4 individuals with another non-leukemia or lymphoma blood cancer ICD-10 recorded). We did not study other cancer sub-types with fewer than 1,000 incident cases to limit multiple hypothesis testing. See **Methods** in S1 File for HES ICD-10 codes for each incident outcome.

### Covariates

Sociodemographic variables were self-reported at the time of assessment, and standard procedures were used to measure height and weight. Highest educational achievement was ranked into: 0 = none, 1 = CSEs (Certificate of Secondary Education), 2 = GCSEs/O-levels (General Certificate of Secondary Education to age 16), 3 = A-levels/NVQ/HND/HNC (further education after age 16), 4 = professional qualification, and 5 = college/university degree. Self-reported cigarette smoking status was coded as never, past or current at assessment. Participants reported frequency and duration of walking, moderate activity and vigorous activity based on the validated International Physical Activity Questionnaire [17], and coded for analysis three groups (low, moderate and vigorous activity).

### Time-to-event analyses

Cox proportional hazards regression (CoxPH) models were used to determine the association between RDW and risk of all-cause mortality, with adjustment for age, sex, smoking status, highest education level attained, mean cell volume (MCV), and circulating hemoglobin levels. For analyses of incident outcomes other than mortality we used Fine and Gray Competing Risk Regression (CRR) analysis, with mortality as the competing risk. All analyses excluded participants with prevalent anemia, CAD, type-2 diabetes, stroke (or TIA), hypertension, COPD, or any cancer (excluding non-melanoma skin cancer; prevalent cancer combined information from self-reports, HES and cancer registry.). Sensitivity analyses adjusting for self-reported physical activity were also performed. Analyses were performed in STATA v14.1, using functions `stcox`and `stcrreg`for CoxPH and CRR models, respectively.

## Results

Of 469,104 UK Biobank participants with complete hematology measures, covariates, and mortality data, we excluded 228,627 participants who had prevalent anemia (self-reported diagnosis, hospital records, or low hemoglobin at the baseline assessment; Hb<120g/L in females, <130g/L in males: from WHO definition [16]), coronary artery disease (CAD), cancer, diabetes, chronic obstructive pulmonary disease (COPD), or hypertension (baseline self-reports or hospital admission electronic records). The analyses therefore included 240,477 apparently disease free UK Biobank participants (Table 1). The baseline mean age of the included sample was 55 years (SD: 8.1, min = 40, max = 70), with 85,352 aged 60 to 70 years old. Males made up 51.8% of the sample, and overall 56.7% reported never having smoked (11.4% were current smokers). Participants were tracked using hospital admission records, with up to 9 years follow up (mean 7 years). In this time 3,888 participants (1.62%) died, and 41,152 (17.1%) had at least 1 hospital admission.

**Table 1:** Characteristics of the 240,477 UK Biobank participants included in analyses.

Trait	N	Mean (SD)	Min—Max
Age (years)	240,477	55.05 (8.1)	40–70
RDW (%)	240,477	13.35 (0.8)	10.8–34.2
Mean Cell Volume (fL)	240,477	91.42 (4.1)	57.4–160.3
Hemoglobin conc. (g/dL)	240,477	144.84 (9.8)	120–206
Follow-up (years since visit)			
*Hospital records* *	240,477	7.00 (0.92)	0.01–9.87
*Death registry*	3,888	4.43 (1.93)	0.06–8.72
Sex	**N**	**%**	
*Females*	115,811	48.16	
*Males*	124,666	51.84	
Smoking status			
*Never*	136,444	56.74	
*Former*	76,707	31.90	
*Current*	27,326	11.36	
Education attained			
*None*	32,440	13.49	
*CSEs (up to 16 years)* †	9,442	3.93	
*GCSEs (up to 16 years)* †	30,648	12.74	
*A-levels (16 to 18 years)* †	45,647	18.98	
*Professional qualification*	35,020	14.56	
*Degree*	87,280	36.29	
RDW (%)			
*<12*.*5*	20,844	8.67	
*12*.*5–12*.*9*	56,446	23.47	
*13*.*0–13*.*4*	73,496	30.56	
*13*.*5–13*.*9*	51,274	21.32	
*14*.*0–14*.*4*	23,600	9.81	
*14*.*5–14*.*9*	8,767	3.65	
≥*15*.*0*	6,050	2.52	

* = years from baseline visit to first hospital admission, or 1st March 2016 if no visit, or death.

^*†*^ = or equivalent qualification; CSEs and GCSEs were lower/higher-level qualifications, respectively

### Survival analysis: RDW is predictive of mortality, cardiovascular disease and cancer

**Fig 1:**
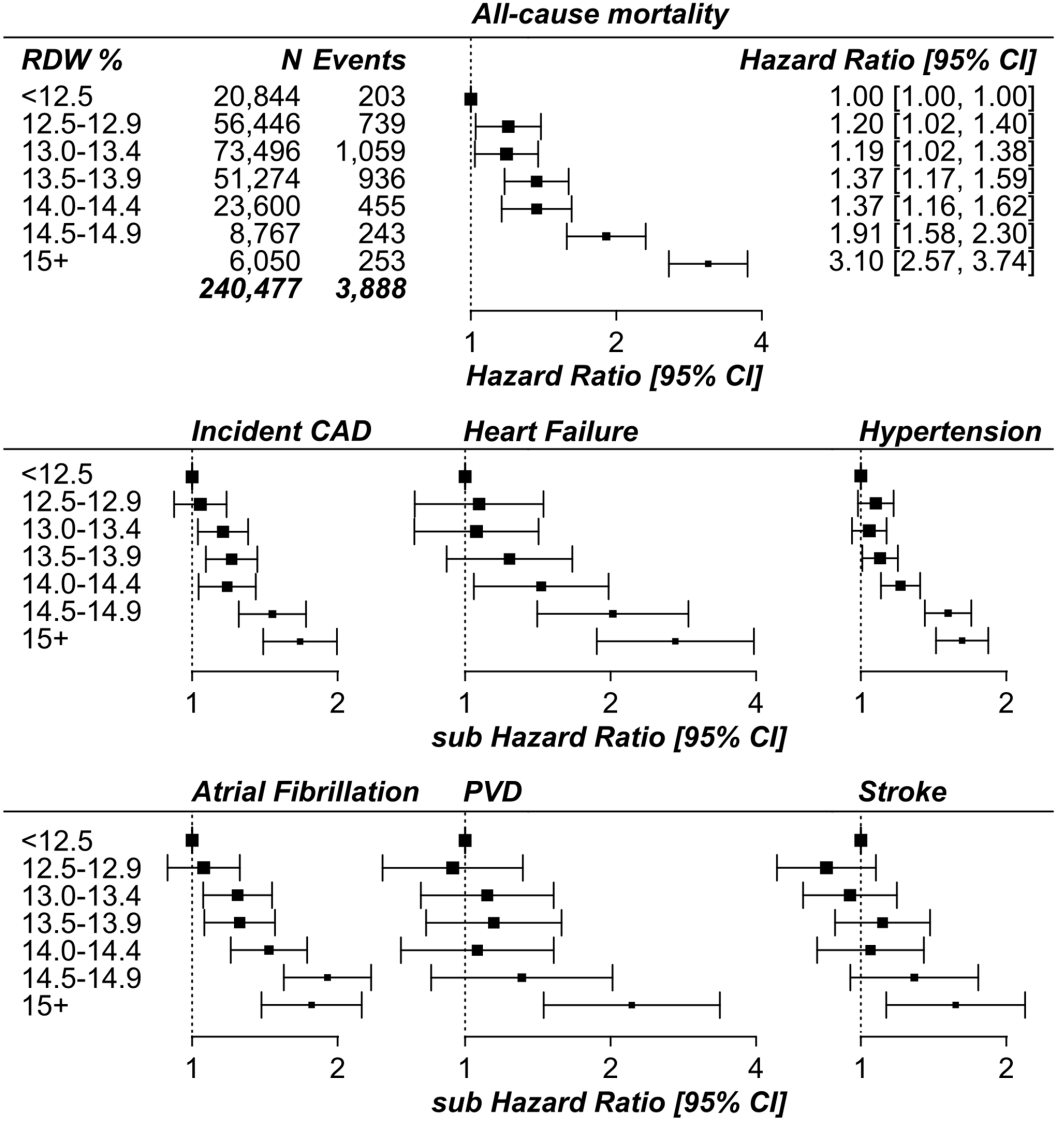
Forest plots showing the Hazard Ratio for each category of RDW from survival analyses. RDW = red blood cell distribution width; CAD = coronary artery disease (myocardial infarction or angina); PVD = peripheral vascular disease; CI = Confidence Interval. All-cause mortality estimates are from Cox Proportional Hazards models; Incident disease estimates are from Competing Risks Regression models (sub Hazard Ratio). Analyses excluded participants with prevalent diseases at baseline assessment (anemia, CAD, cancer, diabetes, stroke, COPD or hypertension), and were adjusted for age, sex, smoking status, education level, hemoglobin concentration, and mean corpuscular volume. See **Table A in** S1 File for full results.

Increasing RDW was positively associated with risk of all-cause mortality in time-to-event analyses adjusted for age, sex, smoking status, education level, hemoglobin concentration, and mean corpuscular volume (Fig 1; **Figure A and Table A in** S1 File). In the multivariate analysis, the risk of death in the highest RDW category (≥15%) compared to the lowest (<12.5%) was greater than the risk in the current smokers compared to the group that never smoked (Hazard Ratio = 3.10: 95% CIs 2.57 to 3.74; HR = 2.46: 95% CIs 2.26 to 2.68, respectively).

In competing risks analysis (where mortality was included as a competing risk) RDW was also separately predictive of incident CAD, heart failure, hypertension, atrial fibrillation, peripheral vascular disease, or stroke (Fig 1 and Table 2; see **Table B in** S1 File for the number of events in each analysis). Additional adjustment for physical activity or BMI did not alter the results, and neither did adjustment for the number of hospitalizations (to determine whether RDW is a risk factor for hospitalization). In combined analysis of the 6 incident cardiovascular outcomes 17,456 participants had at least one diagnosis in the hospital records; those in the highest RDW category (≥15%) compared to the lowest (<12.5%) had 64% increased likelihood of a diagnosis (sub-HR = 1.64: 95% CIs 1.49 to 1.81; **Table A in** S1 File).

**Table 2:** RDW associations with incident conditions in 240,477 UK Biobank participants.

	*12*.*5 to 12*.*9%*			*13 to 13*.*4%*			*13*.*5 to 13*.*9%*			*14 to 14*.*4%*			*14*.*5 to 14*.*9%*			≥*15%*		
	*n* = 56,446			*n* = 73,496			*n* = 51,274			*n* = 23,600			*n* = 8,767			*n* = 6,050		
	HR	95% CIs		HR	95% CIs		HR	95% CIs		HR	95% CIs		HR	95% CIs		HR	95% CIs	
*All-cause mortality*	**1.20**	1.02	1.40	**1.19**	1.02	1.38	**1.20**	1.02	1.40	**1.37**	1.17	1.59	**1.91**	1.58	2.30	**3.10**	2.57	3.74
*Coronary heart disease* *	1.04	0.92	1.18	**1.16**	1.03	1.31	**1.21**	1.07	1.36	**1.18**	1.03	1.35	**1.47**	1.25	1.72	**1.67**	1.40	1.99
*Atrial Fibrillation* *	1.06	0.89	1.26	**1.24**	1.05	1.46	**1.25**	1.06	1.48	**1.44**	1.20	1.73	**1.91**	1.55	2.35	**1.77**	1.39	2.24
*Heart failure* *	1.07	0.79	1.45	1.06	0.79	1.42	1.24	0.92	1.67	**1.44**	1.04	1.98	**2.02**	1.41	2.90	**2.72**	1.87	3.96
*Hypertension* *	1.07	0.99	1.17	1.04	0.96	1.13	**1.10**	1.01	1.19	**1.21**	1.10	1.33	**1.52**	1.36	1.69	**1.62**	1.43	1.83
*Peripheral vascular disease* *	0.94	0.67	1.32	1.11	0.81	1.52	1.15	0.83	1.58	1.06	0.74	1.53	1.31	0.85	2.02	**2.21**	1.45	3.37
*Stroke* *	0.85	0.67	1.07	0.95	0.76	1.19	1.11	0.88	1.39	1.05	0.81	1.35	1.29	0.95	1.75	**1.57**	1.13	2.19
*Cancer (any)* *	1.06	0.97	1.15	1.07	0.99	1.16	**1.10**	1.01	1.19	**1.13**	1.03	1.23	**1.34**	1.20	1.50	**1.37**	1.21	1.55
*Blood cancer (any)* *	0.83	0.61	1.13	1.05	0.78	1.39	1.00	0.74	1.34	1.10	0.79	1.53	**1.69**	1.17	2.46	**2.00**	1.35	2.97
*- Leukemia* *	0.78	0.49	1.26	1.14	0.74	1.78	1.21	0.77	1.90	1.47	0.90	2.38	**2.29**	1.34	3.92	**2.85**	1.63	4.7
*- Lymphoma* *	0.83	0.56	1.22	0.97	0.67	1.39	0.80	0.54	1.18	0.87	0.56	1.34	1.34	0.81	2.19	1.34	0.77	2.34
*Breast cancer* *	1.12	0.95	1.32	1.08	0.92	1.26	1.07	0.90	1.26	1.15	0.95	1.38	1.12	0.87	1.44	1.25	0.93	1.67
*Prostate cancer* *	0.95	0.79	1.14	0.98	0.82	1.17	1.04	0.87	1.24	0.97	0.79	1.18	0.96	0.75	1.23	0.96	0.73	1.27
*Colorectal cancer* *	1.17	0.90	1.51	1.26	0.98	1.61	**1.31**	1.02	1.69	1.29	0.98	1.71	**1.63**	1.18	2.27	**1.92**	1.36	2.72

Analyses exclude participants with prevalent disease at baseline assessment (anemia, CAD, cancer, diabetes, stroke, COPD or hypertension). Adjusted for age, sex, smoking status, educational attainment, hemoglobin and MCV. HR = Hazard Ratio. CIs = Confidence Intervals. Cancer = any cancer, excluding non-melanoma skin cancer. RDW <12.5% is the reference group.

* Competing risks regression models (sub-hazard ratios). Reference group (RDW <12.5%) *n* = 20,844

Of 240,475 participants with available data 11,486 had a new diagnosis of any type of cancer (excluding non-melanoma skin cancer) during follow-up, reported in hospital admission or cancer registry data. RDW was associated with increased risk of cancer, in competing risks analysis (RDW ≥15% sub-HR = 1.37: 95% CIs 1.21 to 1.55; Table 2). We analyzed three sub-types where at least 1,000 incident cases were reported: prostate, breast, and colorectal cancer (n = 2,571, n = 2,323, and n = 1,327, respectively). We also specifically investigated incident cases of blood cancer, any type (n = 839), and specifically leukemia (n = 406) or lymphoma (n = 467). Increased RDW was only associated with incident colorectal cancer (RDW ≥15% sHR = 1.92: 95% CIs 1.36 to 2.72; **Table C in** S1 File) and incident blood cancer (RDW ≥15% sHR = 2.00: 95% CIs 1.35 to 2.97; **Table C in** S1 File). High RDW was only associated specifically with incident leukemia (RDW ≥15% sHR = 2.85: 95% CIs 1.63 to 4.97; **Table C in** S1 File), but not lymphoma (RDW ≥15% sHR = 1.34: 95% CIs 0.77 to 2.34; **S3 Table**). We checked for interactions with mean RBC volume (MCV) and found that the effect of RDW on likelihood of incident colorectal cancer is not altered with higher MCV (interaction p>0.05).

### Stratification of shorter and longer-term events

**Fig 2:**
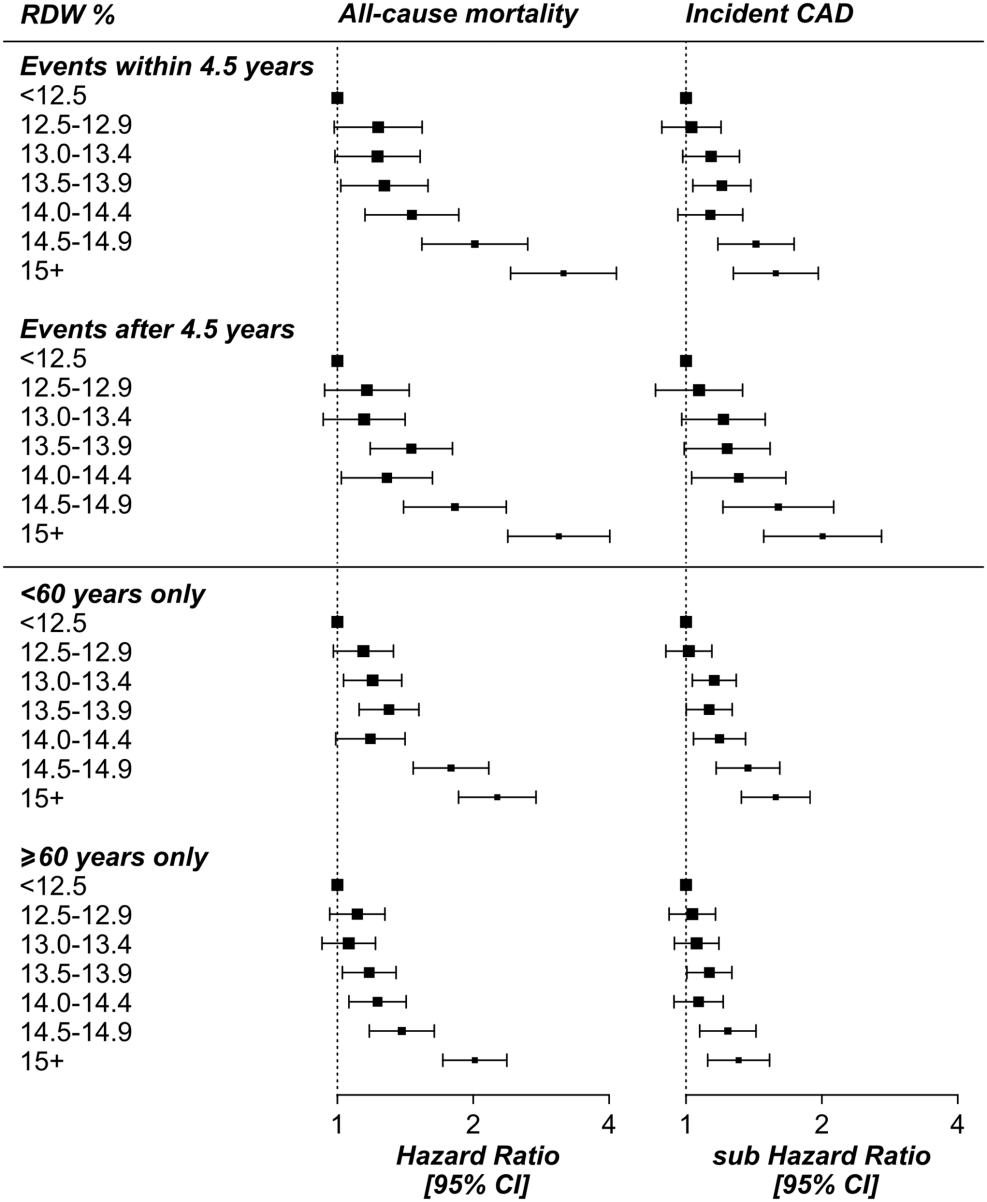
RDW analysis of incident events stratified by follow-up time and age. RDW = red blood cell distribution width; CAD = coronary artery disease (myocardial infarction or angina); CI = Confidence Interval. Hazard Ratios (the mortality analysis) are from Cox Proportional Hazards models; sub Hazard Ratios (for incident disease) are from Competing Risks Regression models. Analyses excluded participants with prevalent diseases at baseline assessment (anemia, CAD, cancer, diabetes, stroke, COPD or hypertension), and were adjusted for age, sex, smoking status, education level, hemoglobin concentration, and mean corpuscular volume. See **Table A in** S1 File for full results.

We stratified the mortality analysis by shorter (1 day to 4.5 years follow-up, i.e. half the max follow-up time of 9 years) and longer-term (4.5 to 9 years) outcomes, to establish whether RDW predictive value was merely short term. There was still strong predictive value when only including longer-term deaths (<4.5 years after assessment HR for ≥15% = 3.17: 95% CIs 2.42 to 4.15; ≥4.5 years HR = 3.09: 95% CIs 2.39 to 4.01) (Fig 2; **Table A in** S1 File). RDW was also predictive of incident cardiovascular conditions after excluding events within 4.5 years of assessment including CAD (sHR = 2.01: 95% CIs 1.49 to 2.71), heart failure (sHR = 2.38: 95% CIs 1.31 to 4.33), hypertension (sHR = 1.57: 95% CIs 1.28 to 1.91), and peripheral vascular disease (sHR = 2.01: 95% CIs 1.49 to 2.71), but not stroke (see **Tables A and B in** S1 File for full details).

The association between RDW and any cancer remained significant after excluding the shorter-term events with 4.5 years of assessment (sHR = 1.42: 1.13 to 1.78) but neither colorectal or leukemia were significantly associated. This may be due to a small number of cases ≥4.5 years after baseline (n = 413 and n = 158, respectively; see **Table D in** S1 File for full details of number of events in each analysis).

### Stratification of analyses by age

The associations between RDW, mortality, and incident events were consistent in analyses restricted to participants aged <60 or to those aged ≥60 (**Table A in** S1 File; Fig 2). Although some associations are no longer significant (e.g. with peripheral vascular disease) this could be due to fewer events in the younger age group (**Table B in** S1 File). All incident outcomes were more common in the older group with the exception of breast cancer: of 2,323 incident diagnoses 1,018 (43.8%) were in those aged ≥60 at baseline, whereas 1,305 (56.2%) were in the younger group. This is in contrast to prostate cancer, where of the 2,571 incident events 1,830 (71.2%) were in those aged ≥60 at baseline.

In some cases the estimate from the whole cohort was greater than the stratified results, for example the Hazard Ratio for RDW% 14.0–14.4 for mortality was 1.37, where the age-stratified Hazard Ratios were 1.29 and 1.36 (Table S1). We performed sensitivity analyses, such as excluding covariates such as age or changing the age cut-off to 55, to check the consistency of this relationship: although the exact association statistics varied we observed the same trend.

## Discussion

In this study we assessed the predictive value of RDW in a large population free of diagnosed disease at baseline, and to assess the prediction of longer-term events only. As expected, we found that increased RDW is strongly predictive of mortality, and incident myocardial infarction. We also found that increased RDW is strongly predictive of increased incidence of hospital recorded heart failure, atrial fibrillation, hypertension, peripheral vascular disease and stroke (independent of number of hospitalizations), suggesting a much wider predictive value for cardiovascular outcomes. To our knowledge this is the first study to make such extensive exclusions of baseline diagnoses to obtain an apparently healthy study group. Although RDW is routinely measured in hematology analyses, it is seldom considered as an informative clinical marker outside of anemia assessment in routine practice.

We questioned whether the predictive value of RDW could be limited to the short term, perhaps as the expression of subclinical pathology; however, the association between high RDW and mortality or incident cardiovascular conditions was confirmed even after excluding incident diagnoses during the first 4.5 years after assessment (half the max follow-up time of 9 years); participants in the highest RDW group (compared to the lowest RDW) were still twice as likely to have a CAD event and 60% more likely to become hypertensive in the period from 4.5 to 9 years after baseline. Only incident stroke diagnoses became non-significant in the longer-term follow-up. This evidence suggests that RDW may be a useful additional marker for cardiovascular (and perhaps colorectal cancer risk), even in primary care and health check settings in people free of disease [12]. In analyses stratified by age (<60 and ≥60 years) the results were consistent, suggesting that, although high RDW is more common in older groups, it is still predictive of incident disease across the 40 to 60 year age-range.

Participants with high RDW were nearly 40% more likely to have any incident cancer; in subset analyses, participants in the highest RDW group were at increased risk of incident leukemia (2.9x) and colorectal cancer (1.9x), compared to those in the lowest group. RDW has previously been reported to be prognostic in patients diagnosed with chronic lymphocytic leukemia [18]. RDW could therefore be reflecting early changes in the bone marrow, where erythrocyte and plasma cell progenitors reside. Regarding colorectal cancer, increased RDW could be due to the bleeding associated with colorectal cancer; a previous study of 115 colon cancer patients found RDW was significantly raised [19]. In our analysis, RDW was only predictive of cancer subtypes in the short term (<4.5 years after assessment), not in the longer term. Breast and prostate cancers–the other two common cancer onsets we were able to study–were not associated. However, both these conditions are seldom directly associated with bleeding.

In comparison to previous studies, our large sample size allowed us to sub-divide RDW into many more categories. For example, the 2013 study by Veeranna *et al* [11] investigating incident cardiovascular deaths split RDW into two groups (≤12.6% and >12.6%); in our analyses we find that the bigger effects are for those with >14% RDW (which includes 16% of the participants), consistent with previous studies of mortality [4]. Many previous studies of RDW were in groups with prevalent diseases; for instance there are strong associations between raised RDW and mortality in participants with CAD [20], PAD [21], pulmonary embolisms [22], or cancer [23], independent of anemia and other prevalent conditions.

Our study is unique for several reasons; we have excluded all prevalent cases of diagnosed cardiovascular disease, cancer and anemia at baseline (including “mild” anemia defined using raised hemoglobin levels) yielding an apparently healthy study group at baseline; we have utilized competing risks regression for incident diseases, yielding estimates that are not biased due to death from other causes (this is particularly important for analyses involving risk factors that predict multiple health outcomes [13]); and we studied associations over 9 years of follow-up. The UK Biobank is a volunteer study with lower all-cause mortality rates than the general population (46% lower in men, and 55% lower in women) of the same age [14], so at assessment the participants were healthier than the general population, strengthening our focus on apparently healthy individuals at baseline.

In a previous study, we found that RDW has a large heritable component (~29%) that increased with age (~34% in individuals aged ≥60 years) [10]. Specific genetic variants for conditions including autoimmune disease, certain cancers, BMI, Alzheimer’s disease, longevity, age at menopause, bone density, myositis, Parkinson’s disease, and age-related macular degeneration were also associated with RDW. However, the majority of variation in RDW was not explained by genetic variants for CAD or related lipid genetic risks, suggesting independent pathways to disease. RDW is simply a measure of the variation in size of RBC, therefore increases in RDW likely reflect impaired erythropoiesis or abnormal survival [6]. There are many possible underlying causes of increased RDW, including inflammation [24], reduced RBC deformability [25], telomere length [6], and others. Our results suggest RDW is not merely a short-term predictor of morbidity and mortality, and could reflect early homeostatic changes.

Future work on vascular outcomes should evaluate the predictive value of RDW in terms of additional information provided above existing cardiovascular risk markers.

### Limitations

The UK Biobank is a healthy volunteer study, with lower all-cause mortality rates than the general population [14]. However, there was substantial variation in RDW within the participants so the results can still be generalized to the wider population [26]. No data have yet been released regarding the UK Biobank participant’s lipid and other blood assays; once this is available, further investigations into the complex relationship between RDW and lipids can be performed. The medical records are currently limited to hospital inpatient data and cancer registry information, meaning that some diagnoses more likely to be made in outpatient or primary care settings could not be studied; however, for the majority of our outcomes such as CAD or stroke, these will be well diagnosed. RDW was available at baseline only, so changes in RDW over time cannot be taken into account in analyses.

### Conclusions

In a large healthy volunteer cohort free of major disease, higher RDW predicted onsets of a wide range of common cardiovascular conditions, plus colorectal cancer. RDW is not merely a short term predictor, but remained predictive 4.5 to 9 years after baseline for most outcomes. RDW may be a useful marker in wellness health check settings in 40 to 70 year olds free of major diagnoses. More work is needed on the mechanisms underlying RDW’s predictive value and to investigate interventions that can modify RDW and give positive effects on health outcomes.

## Supporting information

S1 File Supporting information for this manuscript. **Methods**: ICD-10 diagnosis codes used for incident diseases in the UK Biobank Hospital Episode Statistics. **Figure A**: Kaplan Meier plot showing the survival curve for each category of RDW with mortality. Analysis excluded participants with prevalent diseases (anemia, CAD, cancer, diabetes, stroke, COPD or hypertension). **Table A**: UK Biobank analysis of RBC Distribution Width (RDW) categories and incident mortality and cardiovascular events. Models adjusted for age, sex, smoking, education, MCV and Hemoglobin. Participants with prevalent anemia, CAD, cancer, diabetes, COPD or hypertension were excluded. For mortality Cox’s proportional hazards regression models were used. For CHD, and other incident events competing risks regression models were used (sub Hazard Ratio). **Table B**: Number of events for mortality and cardiovascular outcomes. These are sample sizes for the results in Table A in S1 File. **Table C**: UK Biobank analysis of RBC Distribution Width (RDW) categories and incident cancer. Models adjusted for age, sex, smoking, education, MCV and Hemoglobin. Participants with prevalent anemia, CAD, cancer, diabetes, COPD or hypertension were excluded. Competing risks regression models were used. Analysis of breast cancer includes female participants only. Analysis of prostate cancer includes male participants only. **Table D**: Number of events for cancer outcomes. These are sample sizes for the results in Table C in S1 File.
(DOCX)
