# Supplementary material for: Red cell distribution width and common disease onsets in 240,477 healthy volunteers followed for up to 9 years
Source: PLoS One. 2018 Sep 13;13(9):e0203504. doi: 10.1371/journal.pone.0203504 (PMC6136726; doi:10.1371/journal.pone.0203504)
Supplement: S1 File — Methods: ICD-10 diagnosis codes used for incident diseases in the UK Biobank Hospital Episode Statistics. Figure A: Kaplan Meier plot showing the survival curve for each category of RDW with mortality. Analysis excluded participants with prevalent diseases (anemia, CAD, cancer, diabetes, stroke, COPD or hypertension). Table A: UK Biobank analysis of RBC Distribution Width (RDW) categories and incident mortality and cardiovascular events. Models adjusted for age, sex, smoking, education, MCV and Hemoglobin. Participants with prevalent anemia, CAD, cancer, diabetes, COPD or hypertension were excluded. For mortality Cox’s proportional hazards regression models were used. For CHD, and other incident events competing risks regression models were used (sub Hazard Ratio). Table B: Number of events for mortality and cardiovascular outcomes. These are sample sizes for the results in Table A in S1 File. Table C: UK Biobank analysis of RBC Distribution Width (RDW) categories and incident cancer. Models adjusted for age, sex, smoking, education, MCV and Hemoglobin. Participants with prevalent anemia, CAD, cancer, diabetes, COPD or hypertension were excluded. Competing risks regression models were used. Analysis of breast cancer includes female participants only. Analysis of prostate cancer includes male participants only. Table D: Number of events for cancer outcomes. These are sample sizes for the results in Table C in S1 File. (DOCX) [file pone.0203504.s001.docx]

Pilling *et al.* **Red cell distribution width and common disease onsets in 240,477 healthy volunteers followed for up to 9 years**.

Supporting Information

**Methods – ICD-10 codes for incident diseases:**

Atrial fibrillation: I48

Coronary Artery Disease: I20 - I25

Heart failure: I50; J81

Hypertension: I10 - I15

Peripheral vascular disease: I70.2; I70.9; I73; I74.2; I74.3; I74.4; I74.5; I79.2

Stroke (Cerebral vascular disease): G45 - G46; I61; I63

Blood cancer (any lymphoma, leukemia, other): C81 - C96

Leukemia: C91 - C95

Lymphoma: C81 - C88

**Figure A. Kaplan Meier plot showing the survival curve for each category of RDW with mortality**


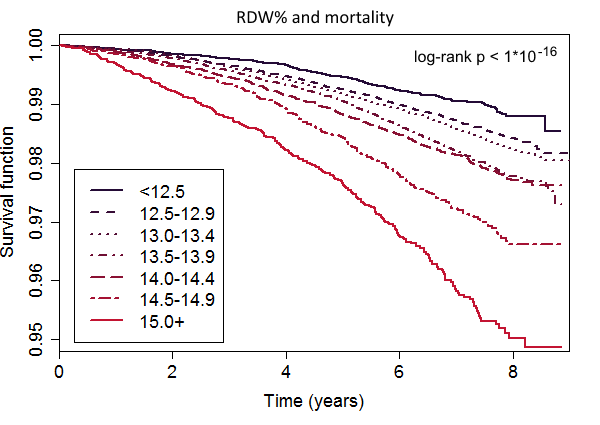


**Table A. UK Biobank analysis of RBC Distribution Width (RDW) categories and incident mortality and cardiovascular events**

|  |  | ***All-cause mortality*** | | | ***Atrial fibrillation*** | | | ***Coronary Artery Disease*** | | | ***Heart failure*** | | | ***Hypertension*** | | | ***Peripheral vascular disease*** | | | ***Stroke (Cerebral vascular disease)*** | | | ***Any of these 6 diseases*** | | |
| --- | --- | --- | --- | --- | --- | --- | --- | --- | --- | --- | --- | --- | --- | --- | --- | --- | --- | --- | --- | --- | --- | --- | --- | --- | --- |
|  | **RDW %** | **HR** | **95% CIs** | | **sHR** | **95% CIs** | | **sHR** | **95% CIs** | | **sHR** | **95% CIs** | | **sHR** | **95% CIs** | | **sHR** | **95% CIs** | | **sHR** | **95% CIs** | | **sHR** | **95% CIs** | |
|  | <12.5 |  |  |  |  |  |  |  |  |  |  |  |  |  |  |  |  |  |  |  |  |  |  |  |  |
| ***All*** | 12.5-12.9 | 1.20 | 1.02 | 1.40 | 1.06 | 0.89 | 1.26 | 1.04 | 0.92 | 1.18 | 1.07 | 0.79 | 1.45 | 1.07 | 0.99 | 1.17 | 0.94 | 0.67 | 1.32 | 0.85 | 0.67 | 1.07 | 1.03 | 0.97 | 1.10 |
| ***participants*** | 13.0-13.4 | 1.19 | 1.02 | 1.38 | 1.24 | 1.05 | 1.46 | 1.16 | 1.03 | 1.31 | 1.06 | 0.79 | 1.42 | 1.04 | 0.96 | 1.13 | 1.11 | 0.81 | 1.52 | 0.95 | 0.76 | 1.19 | 1.06 | 1.00 | 1.14 |
|  | 13.5-13.9 | 1.37 | 1.17 | 1.59 | 1.25 | 1.06 | 1.48 | 1.21 | 1.07 | 1.36 | 1.24 | 0.92 | 1.67 | 1.10 | 1.01 | 1.19 | 1.15 | 0.83 | 1.58 | 1.11 | 0.88 | 1.39 | 1.14 | 1.06 | 1.21 |
|  | 14.0-14.4 | 1.37 | 1.16 | 1.62 | 1.44 | 1.20 | 1.73 | 1.18 | 1.03 | 1.35 | 1.44 | 1.04 | 1.98 | 1.21 | 1.10 | 1.33 | 1.06 | 0.74 | 1.53 | 1.05 | 0.81 | 1.35 | 1.20 | 1.12 | 1.29 |
|  | 14.5-14.9 | 1.91 | 1.58 | 2.30 | 1.91 | 1.55 | 2.35 | 1.47 | 1.25 | 1.72 | 2.02 | 1.41 | 2.90 | 1.52 | 1.36 | 1.69 | 1.31 | 0.85 | 2.02 | 1.29 | 0.95 | 1.75 | 1.48 | 1.36 | 1.62 |
|  | 15.0+ | 3.10 | 2.57 | 3.74 | 1.77 | 1.39 | 2.24 | 1.67 | 1.40 | 1.99 | 2.72 | 1.87 | 3.96 | 1.62 | 1.43 | 1.83 | 2.21 | 1.45 | 3.37 | 1.57 | 1.13 | 2.19 | 1.64 | 1.49 | 1.81 |
|  | <12.5 |  |  |  |  |  |  |  |  |  |  |  |  |  |  |  |  |  |  |  |  |  |  |  |  |
| ***Events*** | 12.5-12.9 | 1.23 | 0.98 | 1.54 | 1.03 | 0.84 | 1.28 | 1.03 | 0.88 | 1.20 | 0.93 | 0.62 | 1.39 | 1.10 | 0.99 | 1.23 | 0.87 | 0.58 | 1.32 | 0.82 | 0.61 | 1.09 | 1.04 | 0.95 | 1.13 |
| ***within 4.5*** | 13.0-13.4 | 1.23 | 0.99 | 1.53 | 1.21 | 0.98 | 1.48 | 1.14 | 0.98 | 1.31 | 1.10 | 0.75 | 1.61 | 1.06 | 0.95 | 1.18 | 1.07 | 0.73 | 1.58 | 0.85 | 0.65 | 1.12 | 1.07 | 0.99 | 1.16 |
| ***years of the*** | 13.5-13.9 | 1.27 | 1.02 | 1.59 | 1.22 | 0.99 | 1.50 | 1.20 | 1.04 | 1.39 | 1.28 | 0.87 | 1.89 | 1.13 | 1.02 | 1.27 | 1.15 | 0.77 | 1.71 | 1.08 | 0.82 | 1.43 | 1.15 | 1.06 | 1.25 |
| ***assessment*** | 14.0-14.4 | 1.46 | 1.15 | 1.86 | 1.32 | 1.05 | 1.66 | 1.13 | 0.96 | 1.34 | 1.61 | 1.07 | 2.44 | 1.22 | 1.08 | 1.38 | 1.01 | 0.64 | 1.58 | 1.02 | 0.75 | 1.39 | 1.19 | 1.08 | 1.30 |
|  | 14.5-14.9 | 2.02 | 1.54 | 2.64 | 1.71 | 1.32 | 2.23 | 1.43 | 1.18 | 1.74 | 2.41 | 1.53 | 3.80 | 1.54 | 1.34 | 1.78 | 1.37 | 0.81 | 2.31 | 1.21 | 0.83 | 1.77 | 1.47 | 1.32 | 1.64 |
|  | 15.0+ | 3.17 | 2.42 | 4.15 | 1.80 | 1.34 | 2.41 | 1.58 | 1.27 | 1.96 | 3.10 | 1.92 | 5.01 | 1.72 | 1.47 | 2.02 | 1.82 | 1.06 | 3.11 | 1.74 | 1.17 | 2.57 | 1.71 | 1.52 | 1.93 |
|  | <12.5 |  |  |  |  |  |  |  |  |  |  |  |  |  |  |  |  |  |  |  |  |  |  |  |  |
| ***Events*** | 12.5-12.9 | 1.16 | 0.94 | 1.44 | 1.10 | 0.82 | 1.47 | 1.07 | 0.86 | 1.33 | 1.27 | 0.80 | 2.03 | 1.04 | 0.91 | 1.19 | 1.07 | 0.86 | 1.33 | 0.92 | 0.61 | 1.38 | 1.03 | 0.92 | 1.15 |
| **after *4.5*** | 13.0-13.4 | 1.15 | 0.93 | 1.41 | 1.31 | 1.00 | 1.73 | 1.21 | 0.98 | 1.50 | 1.00 | 0.63 | 1.59 | 1.01 | 0.89 | 1.15 | 1.21 | 0.98 | 1.50 | 1.15 | 0.78 | 1.69 | 1.05 | 0.95 | 1.17 |
| ***years only*** | 13.5-13.9 | 1.46 | 1.18 | 1.80 | 1.33 | 1.00 | 1.77 | 1.23 | 0.99 | 1.54 | 1.18 | 0.74 | 1.88 | 1.04 | 0.91 | 1.19 | 1.23 | 0.99 | 1.54 | 1.18 | 0.79 | 1.74 | 1.11 | 0.99 | 1.24 |
|  | 14.0-14.4 | 1.29 | 1.02 | 1.62 | 1.69 | 1.25 | 2.29 | 1.31 | 1.03 | 1.66 | 1.21 | 0.73 | 2.02 | 1.21 | 1.04 | 1.40 | 1.31 | 1.03 | 1.66 | 1.12 | 0.72 | 1.74 | 1.24 | 1.10 | 1.40 |
|  | 14.5-14.9 | 1.82 | 1.40 | 2.37 | 2.34 | 1.67 | 3.29 | 1.60 | 1.21 | 2.12 | 1.56 | 0.87 | 2.81 | 1.53 | 1.29 | 1.83 | 1.60 | 1.21 | 2.12 | 1.49 | 0.89 | 2.50 | 1.59 | 1.38 | 1.84 |
|  | 15.0+ | 3.09 | 2.39 | 4.01 | 1.82 | 1.21 | 2.73 | 2.01 | 1.49 | 2.71 | 2.38 | 1.31 | 4.33 | 1.57 | 1.28 | 1.91 | 2.01 | 1.49 | 2.71 | 1.34 | 0.73 | 2.48 | 1.62 | 1.37 | 1.91 |
|  | <12.5 |  |  |  |  |  |  |  |  |  |  |  |  |  |  |  |  |  |  |  |  |  |  |  |  |
| ***Participants*** | 12.5-12.9 | 1.22 | 0.97 | 1.54 | 1.03 | 0.79 | 1.33 | 1.02 | 0.86 | 1.22 | 1.13 | 0.72 | 1.77 | 1.03 | 0.91 | 1.15 | 1.06 | 0.67 | 1.70 | 0.70 | 0.50 | 0.99 | 1.02 | 0.93 | 1.12 |
| ***aged <60*** | 13.0-13.4 | 1.31 | 1.05 | 1.64 | 1.19 | 0.93 | 1.52 | 1.24 | 1.05 | 1.47 | 0.99 | 0.64 | 1.54 | 1.04 | 0.93 | 1.17 | 1.24 | 0.80 | 1.94 | 0.90 | 0.65 | 1.23 | 1.08 | 0.99 | 1.19 |
| ***years only*** | 13.5-13.9 | 1.48 | 1.18 | 1.87 | 1.27 | 0.98 | 1.64 | 1.19 | 1.00 | 1.42 | 1.24 | 0.79 | 1.95 | 1.11 | 0.98 | 1.25 | 1.21 | 0.76 | 1.92 | 0.99 | 0.71 | 1.38 | 1.14 | 1.04 | 1.25 |
|  | 14.0-14.4 | 1.29 | 0.99 | 1.68 | 1.41 | 1.06 | 1.88 | 1.29 | 1.06 | 1.58 | 1.21 | 0.73 | 2.03 | 1.26 | 1.10 | 1.44 | 1.25 | 0.73 | 2.12 | 0.86 | 0.57 | 1.29 | 1.24 | 1.11 | 1.38 |
|  | 14.5-14.9 | 2.39 | 1.79 | 3.19 | 1.76 | 1.24 | 2.50 | 1.61 | 1.26 | 2.05 | 1.99 | 1.11 | 3.57 | 1.69 | 1.43 | 1.99 | 1.84 | 1.00 | 3.41 | 1.23 | 0.75 | 2.01 | 1.63 | 1.43 | 1.86 |
|  | 15.0+ | 3.40 | 2.53 | 4.57 | 1.83 | 1.22 | 2.73 | 1.99 | 1.53 | 2.59 | 2.85 | 1.55 | 5.25 | 2.01 | 1.67 | 2.40 | 3.07 | 1.69 | 5.59 | 1.98 | 1.21 | 3.24 | 1.99 | 1.72 | 2.30 |
|  | <12.5 |  |  |  |  |  |  |  |  |  |  |  |  |  |  |  |  |  |  |  |  |  |  |  |  |
| ***Participants*** | 12.5-12.9 | 1.16 | 0.94 | 1.44 | 1.08 | 0.86 | 1.36 | 1.05 | 0.88 | 1.25 | 1.02 | 0.67 | 1.56 | 1.12 | 0.99 | 1.27 | 0.82 | 0.51 | 1.33 | 0.99 | 0.71 | 1.38 | 1.04 | 0.95 | 1.15 |
| ***aged* ≥*60*** | 13.0-13.4 | 1.09 | 0.89 | 1.34 | 1.28 | 1.03 | 1.60 | 1.08 | 0.91 | 1.29 | 1.09 | 0.73 | 1.63 | 1.04 | 0.93 | 1.17 | 0.98 | 0.63 | 1.53 | 1.01 | 0.74 | 1.39 | 1.05 | 0.95 | 1.15 |
| ***years only*** | 13.5-13.9 | 1.28 | 1.04 | 1.57 | 1.26 | 1.00 | 1.58 | 1.20 | 1.01 | 1.42 | 1.23 | 0.82 | 1.84 | 1.09 | 0.97 | 1.23 | 1.05 | 0.67 | 1.65 | 1.22 | 0.89 | 1.67 | 1.13 | 1.03 | 1.24 |
|  | 14.0-14.4 | 1.36 | 1.09 | 1.69 | 1.47 | 1.16 | 1.87 | 1.10 | 0.91 | 1.33 | 1.54 | 1.01 | 2.36 | 1.18 | 1.04 | 1.35 | 0.90 | 0.54 | 1.49 | 1.19 | 0.84 | 1.68 | 1.17 | 1.06 | 1.30 |
|  | 14.5-14.9 | 1.64 | 1.28 | 2.10 | 1.99 | 1.53 | 2.60 | 1.38 | 1.11 | 1.71 | 2.05 | 1.28 | 3.26 | 1.42 | 1.22 | 1.65 | 0.96 | 0.53 | 1.76 | 1.37 | 0.92 | 2.05 | 1.39 | 1.24 | 1.57 |
|  | 15.0+ | 2.86 | 2.24 | 3.66 | 1.77 | 1.31 | 2.39 | 1.50 | 1.18 | 1.90 | 2.69 | 1.66 | 4.35 | 1.41 | 1.19 | 1.68 | 1.65 | 0.92 | 2.95 | 1.39 | 0.89 | 2.17 | 1.45 | 1.27 | 1.65 |

**Table B. Number of events for mortality and cardiovascular outcomes**

|  | **All** | **Incident** | **Incident** | **Incident** | **Incident** | **Incident** | **Incident** | **Any** |
| --- | --- | --- | --- | --- | --- | --- | --- | --- |
|  | **cause** | **Atrial** | **CAD** | **Heart** | **Hyper-** | **Peripheral** | **Stroke** | **of** |
|  | **mortality** | **Fibrillation** |  | **Failure** | **tension** | **Vascular** |  | **six** |
|  |  |  |  |  |  | **Disease** |  | **diseases** |
| **N** | 240,477 | 238,523 | 239,351 | 240,244 | 239,333 | 239,827 | 240,277 | 240,474 |
| **N events** | 3,888 | 3,080 | 5,430 | 963 | 10,617 | 728 | 1,390 | 17,456 |
| **N competing** | NA | 3,531 | 3,300 | 3,688 | 3,310 | 3,784 | 3,698 | 2,530 |
|  | ***Events within 4.5 years only*** | | | | | | | |
| **N** | 238,450 | 235,568 | 235,869 | 237,931 | 233,561 | 237,600 | 237,849 | 232,127 |
| **N events** | 1,861 | 1,950 | 3,681 | 571 | 6,504 | 471 | 897 | 11,322 |
| **N competing** | NA | 1,705 | 1,567 | 1,767 | 1,651 | 1,814 | 1,763 | 1,252 |
|  | ***Events after 4.5 years only*** | | | | | | | |
| **N** | 238,616 | 234,869 | 234,103 | 237,906 | 231,178 | 237,542 | 237,617 | 226,962 |
| **N events** | 2,027 | 1,130 | 1,749 | 392 | 4,113 | 257 | 493 | 6,134 |
| **N competing** | NA | 1,836 | 1,733 | 1,921 | 1,659 | 1,970 | 1,935 | 1,278 |
|  | ***Participants <60 only*** | | | | | | | |
| **N** | 155,125 | 154,449 | 152,722 | 155,035 | 154,682 | 154,768 | 155,037 | 155,125 |
| **N events** | 1,486 | 1,076 | 2,312 | 344 | 4,529 | 326 | 509 | 7,434 |
| **N competing** | NA | 1,409 | 1,282 | 1,435 | 1,324 | 1,455 | 1,428 | 1,051 |
|  | ***Participants ≥60 only*** | | | | | | | |
| **N** | 85,352 | 84,074 | 84,629 | 85,209 | 84,651 | 85,059 | 85,240 | 85,349 |
| **N events** | 2,402 | 2,004 | 3,118 | 619 | 6,088 | 402 | 881 | 10,022 |
| **N competing** | NA | 2,122 | 2,018 | 2,253 | 1,986 | 2,329 | 2,270 | 1,479 |

**Table C. UK Biobank analysis of RBC Distribution Width (RDW) categories and incident cancer**

|  |  | ***Any incident cancer (excluding NMSC)*** | | | ***Blood cancer (any)*** | | | ***Leukemia*** | | | ***Lymphoma*** | | | ***Breast cancer*** | | | ***Colorectal cancer*** | | | ***Prostate cancer*** | | |
| --- | --- | --- | --- | --- | --- | --- | --- | --- | --- | --- | --- | --- | --- | --- | --- | --- | --- | --- | --- | --- | --- | --- |
|  | **RDW %** | **HR** | **95% CIs** | | **sHR** | **95% CIs** | | **sHR** | **95% CIs** | | **sHR** | **95% CIs** | | **sHR** | **95% CIs** | | **sHR** | **95% CIs** | | **sHR** | **95% CIs** | |
|  | <12.5 |  |  |  |  |  |  |  |  |  |  |  |  |  |  |  |  |  |  |  |  |  |
| ***All*** | 12.5-12.9 | 1.06 | 0.97 | 1.15 | 0.83 | 0.61 | 1.13 | 0.78 | 0.49 | 1.26 | 0.83 | 0.56 | 1.22 | 1.12 | 0.95 | 1.32 | 1.17 | 0.90 | 1.51 | 0.95 | 0.79 | 1.14 |
| ***participants*** | 13.0-13.4 | 1.07 | 0.99 | 1.16 | 1.05 | 0.78 | 1.39 | 1.14 | 0.74 | 1.78 | 0.97 | 0.67 | 1.39 | 1.08 | 0.92 | 1.26 | 1.26 | 0.98 | 1.61 | 0.98 | 0.82 | 1.17 |
|  | 13.5-13.9 | 1.10 | 1.01 | 1.19 | 1.00 | 0.74 | 1.34 | 1.21 | 0.77 | 1.90 | 0.80 | 0.54 | 1.18 | 1.07 | 0.90 | 1.26 | 1.31 | 1.02 | 1.69 | 1.04 | 0.87 | 1.24 |
|  | 14.0-14.4 | 1.13 | 1.03 | 1.23 | 1.10 | 0.79 | 1.53 | 1.47 | 0.90 | 2.38 | 0.87 | 0.56 | 1.35 | 1.15 | 0.95 | 1.38 | 1.29 | 0.98 | 1.71 | 0.97 | 0.79 | 1.18 |
|  | 14.5-14.9 | 1.34 | 1.20 | 1.50 | 1.69 | 1.17 | 2.46 | 2.29 | 1.34 | 3.92 | 1.34 | 0.81 | 2.19 | 1.12 | 0.87 | 1.44 | 1.63 | 1.18 | 2.27 | 0.96 | 0.75 | 1.23 |
|  | 15.0+ | 1.37 | 1.21 | 1.55 | 2.00 | 1.35 | 2.97 | 2.85 | 1.63 | 4.97 | 1.34 | 0.77 | 2.34 | 1.25 | 0.93 | 1.67 | 1.92 | 1.36 | 2.72 | 0.96 | 0.73 | 1.27 |
|  | <12.5 |  |  |  |  |  |  |  |  |  |  |  |  |  |  |  |  |  |  |  |  |  |
| ***Events*** | 12.5-12.9 | 1.05 | 0.95 | 1.15 | 0.74 | 0.51 | 1.07 | 0.69 | 0.37 | 1.28 | 0.72 | 0.47 | 1.12 | 1.12 | 0.93 | 1.36 | 1.25 | 0.90 | 1.72 | 0.88 | 0.71 | 1.09 |
| ***within 4.5*** | 13.0-13.4 | 1.07 | 0.97 | 1.18 | 0.95 | 0.68 | 1.34 | 1.22 | 0.70 | 2.13 | 0.78 | 0.51 | 1.18 | 1.05 | 0.87 | 1.26 | 1.28 | 0.94 | 1.75 | 0.97 | 0.79 | 1.19 |
| ***years of the*** | 13.5-13.9 | 1.07 | 0.97 | 1.18 | 0.81 | 0.57 | 1.16 | 1.12 | 0.63 | 1.99 | 0.61 | 0.39 | 0.95 | 1.01 | 0.83 | 1.23 | 1.55 | 1.13 | 2.12 | 0.98 | 0.79 | 1.21 |
| ***assessment*** | 14.0-14.4 | 1.12 | 1.01 | 1.25 | 1.03 | 0.69 | 1.52 | 1.59 | 0.86 | 2.93 | 0.80 | 0.49 | 1.32 | 1.12 | 0.89 | 1.39 | 1.39 | 0.99 | 1.97 | 0.90 | 0.71 | 1.14 |
|  | 14.5-14.9 | 1.32 | 1.16 | 1.51 | 1.67 | 1.08 | 2.59 | 2.19 | 1.09 | 4.37 | 1.34 | 0.77 | 2.34 | 1.19 | 0.89 | 1.59 | 1.88 | 1.26 | 2.80 | 0.92 | 0.69 | 1.24 |
|  | 15.0+ | 1.38 | 1.19 | 1.61 | 2.02 | 1.27 | 3.22 | 3.47 | 1.75 | 6.88 | 1.15 | 0.60 | 2.22 | 1.13 | 0.80 | 1.61 | 2.24 | 1.47 | 3.40 | 0.84 | 0.60 | 1.18 |
|  | <12.5 |  |  |  |  |  |  |  |  |  |  |  |  |  |  |  |  |  |  |  |  |  |
| ***Events*** | 12.5-12.9 | 1.08 | 0.94 | 1.25 | 1.09 | 0.61 | 1.94 | 0.94 | 0.44 | 2.01 | 1.27 | 0.55 | 2.92 | 1.12 | 0.83 | 1.52 | 1.04 | 0.68 | 1.59 | 1.12 | 0.80 | 1.58 |
| ***after 4.5 years*** | 13.0-13.4 | 1.09 | 0.94 | 1.25 | 1.31 | 0.75 | 2.26 | 1.03 | 0.50 | 2.12 | 1.74 | 0.79 | 3.83 | 1.17 | 0.87 | 1.57 | 1.23 | 0.82 | 1.84 | 0.99 | 0.71 | 1.38 |
| ***only*** | 13.5-13.9 | 1.18 | 1.02 | 1.36 | 1.51 | 0.86 | 2.63 | 1.35 | 0.65 | 2.79 | 1.61 | 0.72 | 3.64 | 1.23 | 0.90 | 1.68 | 0.91 | 0.59 | 1.40 | 1.20 | 0.86 | 1.68 |
|  | 14.0-14.4 | 1.14 | 0.97 | 1.35 | 1.32 | 0.71 | 2.45 | 1.29 | 0.58 | 2.86 | 1.16 | 0.46 | 2.93 | 1.22 | 0.86 | 1.75 | 1.13 | 0.70 | 1.81 | 1.17 | 0.81 | 1.69 |
|  | 14.5-14.9 | 1.42 | 1.17 | 1.73 | 1.81 | 0.90 | 3.66 | 2.46 | 1.05 | 5.75 | 1.38 | 0.46 | 4.15 | 0.91 | 0.54 | 1.54 | 1.25 | 0.69 | 2.24 | 1.12 | 0.71 | 1.76 |
|  | 15.0+ | 1.42 | 1.13 | 1.78 | 2.06 | 0.97 | 4.36 | 1.96 | 0.74 | 5.20 | 2.24 | 0.76 | 6.58 | 1.62 | 0.96 | 2.72 | 1.46 | 0.76 | 2.79 | 1.39 | 0.86 | 2.27 |
|  | <12.5 |  |  |  |  |  |  |  |  |  |  |  |  |  |  |  |  |  |  |  |  |  |
| ***Participants*** | 12.5-12.9 | 1.08 | 0.97 | 1.21 | 0.74 | 0.49 | 1.11 | 0.59 | 0.31 | 1.12 | 0.84 | 0.50 | 1.43 | 1.06 | 0.87 | 1.29 | 1.17 | 0.83 | 1.66 | 1.02 | 0.73 | 1.42 |
| ***aged <60*** | 13.0-13.4 | 1.12 | 1.01 | 1.25 | 0.93 | 0.64 | 1.37 | 0.98 | 0.55 | 1.75 | 0.86 | 0.52 | 1.43 | 1.06 | 0.87 | 1.29 | 1.14 | 0.81 | 1.60 | 1.26 | 0.92 | 1.72 |
| ***years only*** | 13.5-13.9 | 1.14 | 1.01 | 1.27 | 0.86 | 0.57 | 1.30 | 0.92 | 0.49 | 1.70 | 0.79 | 0.46 | 1.37 | 1.07 | 0.87 | 1.32 | 1.23 | 0.86 | 1.75 | 1.37 | 1.00 | 1.89 |
|  | 14.0-14.4 | 1.13 | 0.99 | 1.29 | 0.96 | 0.60 | 1.55 | 1.32 | 0.67 | 2.58 | 0.80 | 0.42 | 1.52 | 1.11 | 0.87 | 1.42 | 1.03 | 0.67 | 1.58 | 1.09 | 0.75 | 1.59 |
|  | 14.5-14.9 | 1.44 | 1.22 | 1.70 | 1.39 | 0.79 | 2.46 | 1.98 | 0.90 | 4.35 | 1.04 | 0.47 | 2.30 | 0.90 | 0.62 | 1.29 | 1.86 | 1.15 | 3.00 | 0.93 | 0.55 | 1.58 |
|  | 15.0+ | 1.43 | 1.18 | 1.73 | 1.77 | 0.98 | 3.20 | 2.86 | 1.29 | 6.37 | 1.00 | 0.41 | 2.43 | 1.24 | 0.85 | 1.81 | 1.68 | 0.96 | 2.92 | 1.46 | 0.87 | 2.44 |
|  | <12.5 |  |  |  |  |  |  |  |  |  |  |  |  |  |  |  |  |  |  |  |  |  |
| ***Participants*** | 12.5-12.9 | 1.04 | 0.92 | 1.16 | 0.97 | 0.61 | 1.55 | 1.09 | 0.52 | 2.27 | 0.82 | 0.46 | 1.46 | 1.24 | 0.94 | 1.63 | 1.19 | 0.81 | 1.74 | 0.90 | 0.72 | 1.12 |
| ***aged* ≥*60*** | 13.0-13.4 | 1.03 | 0.92 | 1.16 | 1.20 | 0.77 | 1.87 | 1.42 | 0.71 | 2.86 | 1.06 | 0.62 | 1.82 | 1.13 | 0.86 | 1.48 | 1.40 | 0.97 | 2.02 | 0.85 | 0.69 | 1.05 |
| ***years only*** | 13.5-13.9 | 1.06 | 0.95 | 1.19 | 1.17 | 0.74 | 1.83 | 1.62 | 0.80 | 3.26 | 0.82 | 0.47 | 1.43 | 1.09 | 0.83 | 1.44 | 1.43 | 0.99 | 2.06 | 0.90 | 0.73 | 1.12 |
|  | 14.0-14.4 | 1.10 | 0.98 | 1.25 | 1.28 | 0.79 | 2.08 | 1.79 | 0.86 | 3.74 | 0.94 | 0.51 | 1.72 | 1.21 | 0.90 | 1.65 | 1.51 | 1.02 | 2.24 | 0.90 | 0.71 | 1.13 |
|  | 14.5-14.9 | 1.26 | 1.09 | 1.46 | 2.04 | 1.21 | 3.43 | 2.87 | 1.31 | 6.30 | 1.56 | 0.80 | 3.03 | 1.39 | 0.96 | 2.01 | 1.58 | 1.00 | 2.49 | 0.93 | 0.70 | 1.24 |
|  | 15.0+ | 1.30 | 1.10 | 1.53 | 2.35 | 1.35 | 4.09 | 3.28 | 1.44 | 7.46 | 1.64 | 0.79 | 3.41 | 1.27 | 0.80 | 2.02 | 2.16 | 1.35 | 3.44 | 0.82 | 0.59 | 1.14 |

**Table D. Number of events for cancer outcomes**

|  | **Incident** | **Incident** | **Incident** | **Incident** | **Incident** | **Incident** | **Incident** |
| --- | --- | --- | --- | --- | --- | --- | --- |
|  | **cancer** | **blood** | **leukemia** | **lymphoma** | **breast** | **colorectal** | **prostate** |
|  | **(any)** | **cancer** |  |  | **cancer** | **cancer** | **cancer** |
| **N** | 240,475 | 240,477 | 240,477 | 240,477 | 115,811 | 240,477 | 124,666 |
| **N events** | 11,486 | 839 | 406 | 467 | 2,304 | 1,327 | 2,567 |
| **N competing** | 1,405 | 3,669 | 3,767 | 3,777 | 1,223 | 3,547 | 2,405 |
|  | ***Events within 4.5 years only*** | | | | | | |
| **N** | 236,190 | 238,300 | 240,477 | 238,374 | 114,502 | 238,216 | 122,666 |
| **N events** | 7,883 | 550 | 406 | 317 | 1,636 | 914 | 1,791 |
| **N competing** | 721 | 1,781 | 3,767 | 1,824 | 582 | 1,699 | 1,181 |
|  | ***Events after 4.5 years only*** | | | | | | |
| **N** | 231,873 | 238,146 | 238,413 | 238,336 | 113,593 | 237,864 | 121,694 |
| **N events** | 3,603 | 289 | 158 | 150 | 668 | 413 | 776 |
| **N competing** | 684 | 1,888 | 1,951 | 1,953 | 641 | 1,848 | 1,224 |
|  | ***Participants <60 only*** | | | | | | |
| **N** | 155,125 | 155,125 | 155,125 | 155,125 | 72,940 | 155,125 | 82,155 |
| **N events** | 4,934 | 355 | 164 | 200 | 1,296 | 545 | 739 |
| **N competing** | 582 | 1,415 | 1,446 | 1,450 | 459 | 1,348 | 945 |
|  | ***Participants* ≥*60 only*** | | | | | | |
| **N** | 85,350 | 85,352 | 85,352 | 85,352 | 42,841 | 85,352 | 42,511 |
| **N events** | 6,552 | 484 | 242 | 267 | 1,008 | 782 | 1,828 |
| **N competing** | 823 | 2,254 | 2,321 | 2,327 | 764 | 2,199 | 1,460 |
